# Diagnostic accuracy of procalcitonin, neutrophil-lymphocyte count ratio, C-reactive protein, and lactate in patients with suspected bacterial sepsis

**DOI:** 10.1371/journal.pone.0181704

**Published:** 2017-07-20

**Authors:** Lars Ljungström, Anna-Karin Pernestig, Gunnar Jacobsson, Rune Andersson, Barbara Usener, Diana Tilevik

**Affiliations:** 1 Department of Infectious Diseases, Skaraborg Hospital, Skövde, Sweden; 2 Systems Biology Research Centre, School of Bioscience, University of Skövde, Skövde, Sweden; 3 CARe–Center for Antibiotic Resistance Research, Gothenburg University, Gothenburg, Sweden; 4 Department of Infectious Diseases, Institute of Biomedicine, Sahlgrenska Academy, Gothenburg University and Sahlgrenska University Hospital, Gothenburg, Sweden; 5 Department of Clinical Chemistry, Unilabs AB, Skövde, Sweden; Hospital Sirio-Libanes, BRAZIL

## Abstract

**Background:**

Early recognition is a key factor to achieve improved outcomes for septic patients. Combinations of biomarkers, as opposed to single ones, may improve timely diagnosis and survival. We investigated the performance characteristics of sepsis biomarkers, alone and in combination, for diagnosis of verified bacterial sepsis using Sepsis-2 and Sepsis-3 criteria, respectively.

**Methods:**

Procalcitonin (PCT), neutrophil-lymphocyte count ratio (NLCR), C-reactive protein (CRP), and lactate were determined in a total of 1,572 episodes of adult patients admitted to the emergency department on suspicion of sepsis. All sampling were performed prior to antibiotic administration. Discriminant analysis was used to construct two composite biomarkers consisting of linear combinations of the investigated biomarkers, one including three selected biomarkers (i.e., NLCR, CRP, and lactate), and another including all four (i.e., PCT, NLCR, CRP, and lactate). The diagnostic performances of the composite biomarkers as well as the individual biomarkers were compared using the area under the receiver operating characteristic curve (AUC).

**Results:**

For diagnosis of bacterial sepsis based on Sepsis-3 criteria, the AUC for PCT (0.68; 95% CI 0.65–0.71) was comparable to the AUCs for the both composite biomarkers. Using the Sepsis-2 criteria for bacterial sepsis diagnosis, the AUC for the NLCR (0.68; 95% CI 0.65–0.71) but not for the other single biomarkers, was equal to the AUCs for the both composite biomarkers. For diagnosis of severe bacterial sepsis or septic shock based on the Sepsis-2 criteria, the AUCs for both composite biomarkers were significantly greater than those of the single biomarkers (0.85; 95% CI 0.82–0.88 for the composite three-biomarker, and 0.86; 95% CI 0.83–0.89 for the composite four-biomarker).

**Conclusions:**

Combinations of biomarkers can improve the diagnosis of verified bacterial sepsis in the most critically ill patients, but in less severe septic conditions either the NLCR or PCT alone exhibit equivalent performance.

## Introduction

Sepsis is a life-threatening condition that arises when the host responds to an infection and damages its organs [1]. It is often difficult to distinguish between bacterial and non-bacterial aetiologies early in suspected sepsis. Clinical signs of sepsis such as tachycardia, leucocytosis, tachypnea, and pyrexia, often overlap with other non-infectious conditions in critically ill patients. Concurrently, it has been shown that prompt diagnosis and early administration of appropriate antibiotic therapy considerably improve the outcomes of septic patients [2–4]. The difficulty in distinguishing between bacterial and non-bacterial aetiologies is also a major cause of the misuse of antibiotics [5]. Inappropriate or prolonged use of antibiotics may lead to the emergence of antibiotic resistant bacteria [6–8] and to various adverse events [9–11], whereas antibiotic underuse due to delayed or missed diagnosis may result in worsened condition and medical complications [12–16].

There is an unmet need for diagnostic tools differentiating between bacterial and non-bacterial causes of sepsis. Although various biomarkers have been proposed, no single clinical or biological indicator of sepsis has gained general acceptance [17, 18]. The most widely studied biomarkers in patients with suspected bacterial sepsis are C-reactive protein (CRP) and procalcitonin (PCT). Both CRP and PCT are today routinely employed in clinical practice but have limited abilities to distinguish bacterial sepsis from other inflammatory conditions [19]. Even if PCT has an established role as biomarker in septic patients, the diagnostic accuracy of routine PCT measurements have been questioned because of inconsistent and variable results depending on the severity of illness and infection in the studied patient population [20–38]. Lactate is another biomarker frequently used as a biomarker in septic patients, however, it lacks specificity [39] as elevated lactate levels can be seen in a wide variety of conditions in addition to sepsis, e.g., cardiac arrest, trauma, seizure [40]. Zahorec et al. were the first to propose to use the ratio of neutrophil and lymphocyte count (NLCR) as an additional infection marker in clinical practice [41]. Previously, the NLCR has been found to correlate with disease severity [41–43] and has also been suggested as a predictor of bacteraemia [44–48]. Considering the complexity of sepsis, no single marker is good enough for precise diagnostics, but a combination of biomarkers could improve diagnosis [49] and treatment efficacy, and patient outcome.

In view of previously results and the paramount importance of timely diagnosis and accurate treatment of bacterial sepsis, we undertook the present study to determine the diagnostic value of PCT and the NLCR in comparisons with two conventional biomarkers, i.e., CRP and lactate, using a large sample size. In addition, we determined the discriminatory power of combining multiple biomarkers in diagnosis of adult patients suspected of having community-onset sepsis.

## Materials and methods

### Patients and study design

This study is part of a prospective observational study of community-onset severe sepsis and septic shock in adults conducted from September 2011 to June 2012 at Skaraborg Hospital, a secondary hospital with 640 beds, in the western region of Sweden. All patients ≥18 years consecutively admitted to the emergency department for suspicion of a community-onset sepsis were asked to participate in the study. Only those patients who gave their written informed consent were enrolled. The study was approved by the Regional Ethical Review Board of Gothenburg (376–11).

**Fig 1:**
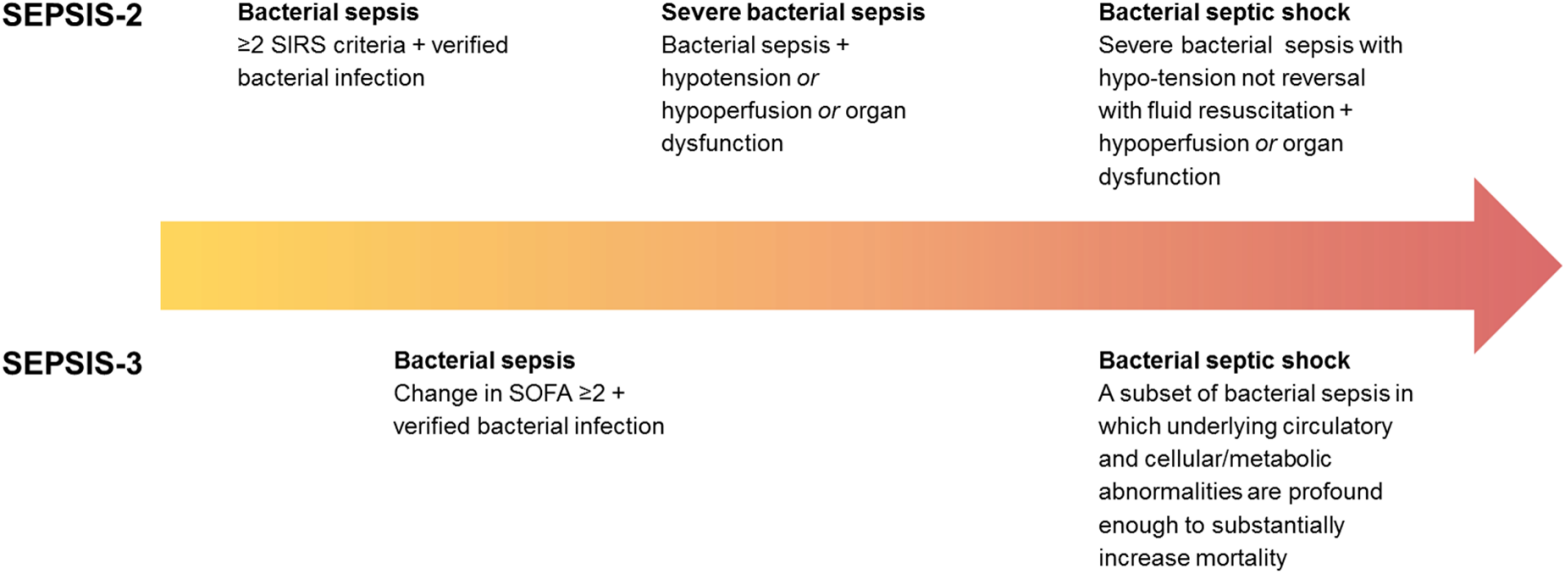
Definitions of verified bacterial sepsis, severe bacterial sepsis, and bacterial septic shock according to Sepsis-2 and Sepsis-3, respectively. SIRS, systemic inflammatory response syndrome; MAP, mean arterial pressure.

At the time of admission to the emergency department, signs and symptoms, clinical and laboratory data were collected and recorded. The sampling was performed according to routine hospital procedures and prior to administration of antibiotic therapy. All medical records were retrospectively reviewed by two senior specialists in infectious diseases (LL and GJ) to determine whether the patients fulfilled Sepsis-2 and Sepsis-3 criteria, respectively. According to Sepsis-2 criteria, bacterial sepsis was defined as verified bacterial infection and systemic inflammatory response syndrome (SIRS), whereas severe bacterial sepsis was defined as verified bacterial sepsis and sepsis-induced hypotension or tissue hypoperfusion or organ dysfunction according to the Swedish criteria (Fig 1; S1 Text) [50]. SIRS was defined as the occurrence of at least two of the following criteria: fever >38.0^°^C or hypothermia <36.0^°^C, tachypnea >20 breaths/min, tachycardia >90 beats/min, leucocytosis >12x10^9^ cells/L or leucopenia <4x10^9^ cells/L. According to Sepsis-3 criteria, bacterial sepsis was defined as bacterial infection-induced organ dysfunction characterized by a rise in total SOFA ≥2 (Fig 1) [1]. For both Sepsis-2 and Sepsis-3, verified bacterial infection was defined as a clinical infection and identification of relevant bacteria by culture, or as typical clinical symptoms, such as erysipelas. Bacteraemia was defined as a positive blood culture result.

### Biomarker measurements

CRP levels were measured with the ADVIA Chemistry Instrument (Siemens Healthcare Diagnostics Inc.) and plasma lactate on an ABL 800 Flex (Radiometer Medical ApS). NLCR were determined on an ADVIA 2120i (Siemens Healthcare Diagnostics Inc.), counting neutrophils and lymphocytes using the white blood cell differential methods to calculate the NLCR. CRP, lactate and NLCR were analysed on the same day as collected and as per manufacturer´s instructions. For the PCT measurements, samples were taken into sodium citrate tubes, centrifuged and plasma stored at -80^°^C until the end of study when all samples were retrospectively measured on a mini-VIDAS® (bioMérieux, France) as per the manufacturer’s instructions.

### Statistical analysis

The continuous variables were expressed as the median and interquartile range due to non-normal distribution (*p* <0.05 for Kolmogorov-Smirnov´s test for all continuous variables). The categorical variables were summarised as frequencies and percentages. Mann-Whitney U tests were performed for comparisons of continuous variables in different patient groups. Adjustment of *p* values for multiple comparisons were made using the Benjamini and Hochberg method. For the diagnostic evaluation of single biomarkers, the performance characteristics for several cut-off points were recorded. The different cut-offs were selected for each biomarker based on previously reported findings as well as recommendations for clinical practice in Sweden [44, 51–53]. Two composite biomarkers were constructed using linear discriminant analysis (S2 Text). One composite biomarker consisted of a linear combination of three selected biomarkers, and the other one included all four biomarkers. The three-marker combination consisted of CRP, lactate, and NLCR, and were primarily chosen as they are routinely employed in our hospital and can easily be integrated in daily practice without extra costs. A comparison of the diagnostic accuracy of the biomarkers, alone and in combination, was made using receiver operating characteristics curves (ROC) analyses by calculating the area under the curve (AUC). For comparison of AUCs, DeLong's test for two correlated ROC curves was used [54, 55]. All tests were two-sided, and *p* <0.05 was considered statistically significant. The statistical analyses were performed using IBM SPSS Statistics version 24.0 (IBM Corp., United States), R version 3.2.3 (R Foundation for Statistical Computing, Austria), and MATLAB R2016a (The Mathworks Inc., United States).

## Results

### Patients

**Fig 2:**
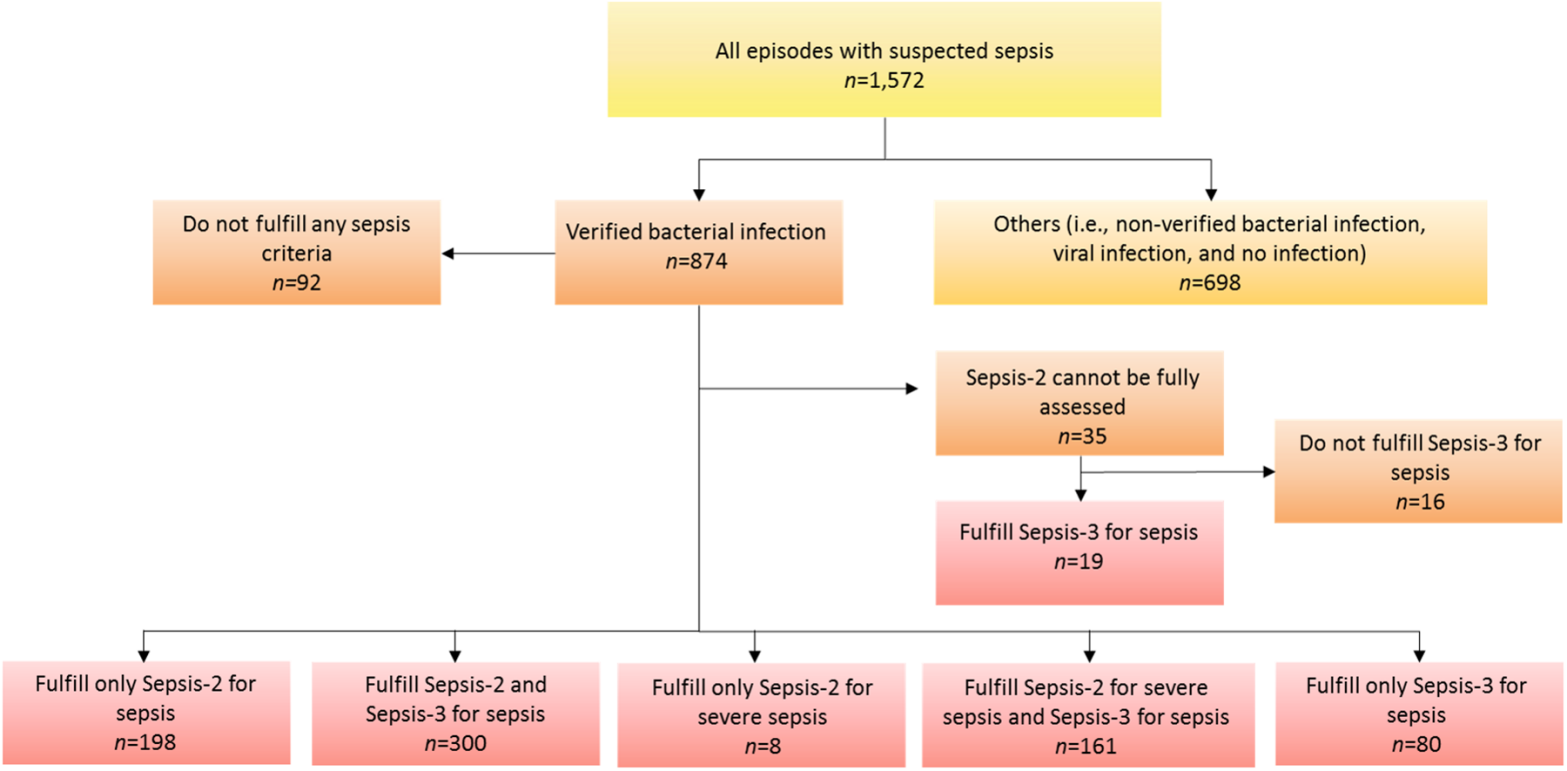
Distribution of 1,572 episodes of adult patients with suspected sepsis depending on presence of bacterial infection and whether the criteria for Sepsis-2 and/or Sepsis-3 were fulfilled.

**Fig 3:**
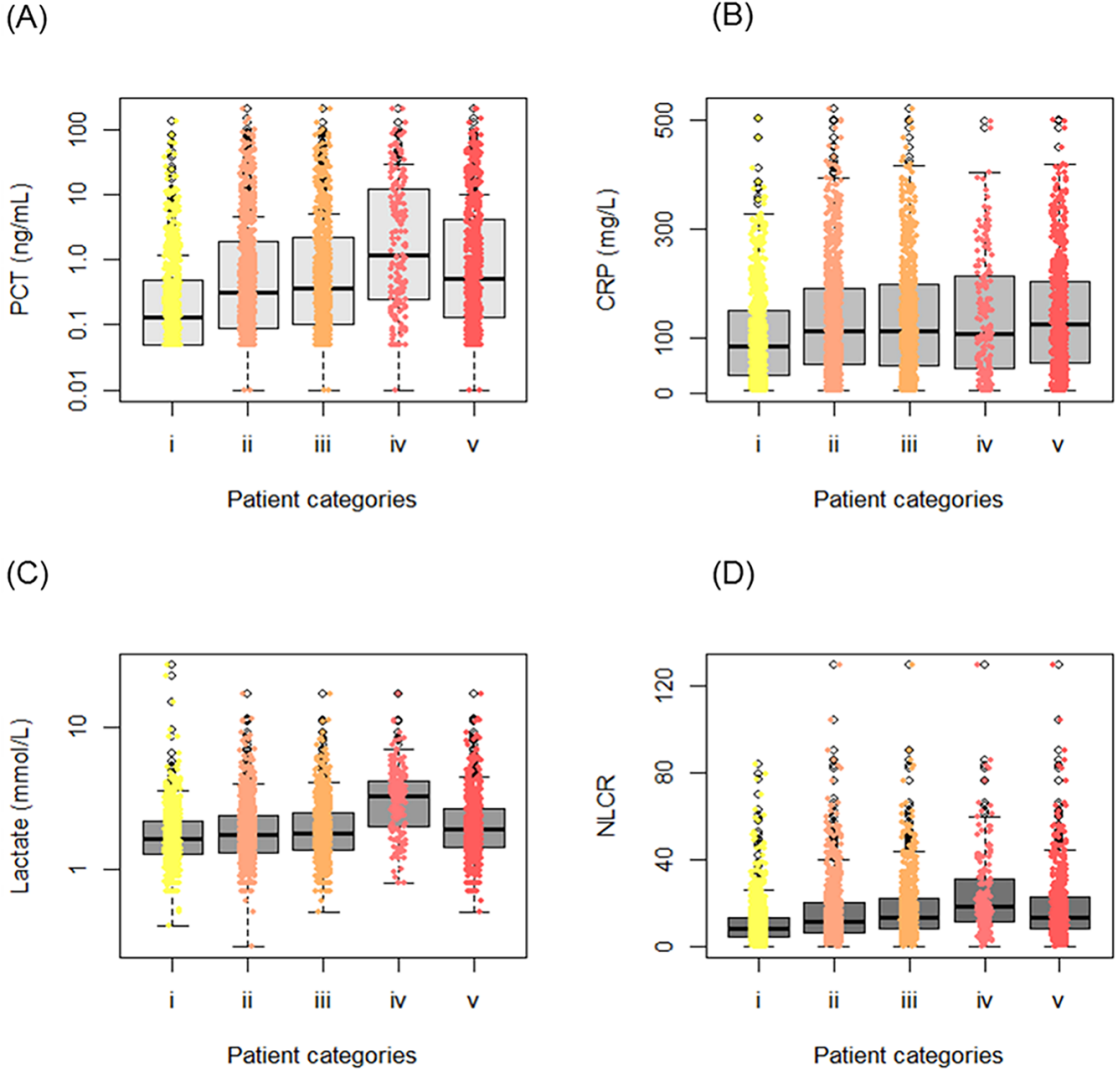
Comparisons of levels of single biomarkers stratified by patient categories. i. Others (i.e., non-verified bacterial infection, viral infection, and no infection) (*n* = 698); ii. Verified bacterial infection (*n* = 874); iii. Verified bacterial sepsis (Sepsis-2) (*n* = 667); iv. Verified severe bacterial sepsis/septic shock (Sepsis-2) (*n* = 169); v. Verified bacterial sepsis (Sepsis-3) (*n* = 560). (A) Procalcitonin (PCT), (B) C-reactive protein (CRP), (C) Lactate, (D) Neutrophil-lymphocyte count ratio (NLCR).

A total of 1,572 episodes of adult patients suspected with sepsis admitted to the emergency department at Skaraborg Hospital were enrolled. Of these, 874 episodes (55.6%) had a verified bacterial infection (Fig 2), whereas the remaining 698 episodes did not. Six-hundred and sixty-seven episodes (42.4%) fulfilled the Sepsis-2 criteria for bacterial sepsis, whereof 169 episodes (10.8%) also fulfilled the criteria for severe bacterial sepsis or septic shock. For 35 of the episodes having a bacterial infection, at least one of four SIRS criteria was missing and thus not possible to evaluate based on the Sepsis-2 criteria. Five-hundred and sixty episodes (35.6%) fulfilled the Sepsis-3 criteria for bacterial sepsis. Patient characteristics by categories are summarised in Table 1, whereas the levels of the biomarkers by patient categories are shown in Fig 3.

**Table 1:** Patient characteristics at admission.

				Sepsis-2		Sepsis-3
Characteristic	All episodes suspected with sepsis (*n* = 1,572)	Others^a^ (*n* = 698)	Bacterial infection (*n* = 874)	Bacterial sepsis^b^ (*n* = 667)	Severe bacterial sepsis/septic shock (*n* = 169)	Bacterial sepsis^c^ (*n* = 560)
**Age (years)**	71 (58–81)	70 (56–80)	73 (59–81)	73 (60–82)	76 (68–84)	77 (67–85)
**Sex (female)**	697 (44.3%)	297 (42.6%)	400 (45.8%)	305 (45.7%)	76 (45.0%)	254 (45.4%)
**Systolic blood pressure (mmHg)**	134 (118–150)	138 (120–154)	131 (115–150)	131 (114–150)	120.0 (104–142)	130 (111–150)
**Respiratory rate (breaths/min)**	24 (20–28)	23 (18–28)	24 (20–28)	24 (22–30)	28 (24–32)	25 (21–30)
**Oxygen saturation (%)**	95 (92–97)	95 (92–97)	95 (92–97)	95 (92–96)	93 (88–96)	94 (90–96)
**Heart rate (beats/min)**	96 (83–109)	96 (82–108)	97 (84–110)	102 (91–114)	108 (94–121)	99 (85–113)
**Body temperature (**^**o**^**C)**	37.9 (37.1–38.6)	37.8 (37.0–38.5)	38.0 (37.2–38.7)	38.3 (37.5–38.8)	38.2 (37.4–38.8)	38.0 (37.1–38.7)
**Haemoglobin (g/L)**	131 (118–142)	132 (119–144)	130 (117–141)	130 (118–141)	130 (117–143)	128 (116–140)
**LPK (x10**^**9**^ **cells/L)**	12.0 (8.7–15.6)	11.1 (7.8–14.5)	12.5 (9.4–16.5)	13.6 (10.3–17.7)	14.7 (10.5–19.3)	13.1 (9.8–17.6)
**CRP (mg/L)**	102 (43–174)	85 (31–152)	114 (51–191)	114 (51–198)	109 (43–216)	126 (54–204)
**PCT (ng/mL)**	0.20 (0.06–1.07)	0.13 (0.05–0.49)	0.31 (0.09–1.87)	0.37 (0.10–2.17)	1.17 (0.24–12.40)	0.51 (0.13–4.07)
**P-lactate (mmol/L)**	1.70 (1.30–2.31)	1.63 (1.27–2.20)	1.77 (1.30–2.40)	1.81 (1.37–2.49)	3.30 (2.00–4.20)	1.90 (1.44–2.70)
**NLCR**	9.6 (5.4–16.8)	7.8 (4.4–13.0)	11.5 (6.5–19.9)	13.1 (7.9–22.1)	18.4 (11.2–31.2)	13.0 (8.0–22.4)
**ICU (yes)**	111 (7.1%)	52 (7.4%)	59 (6.8%)	52 (7.8%)	46 (27.2%)	59 (10.5%)
**28 days survival**	1489 (94.7%)	650 (93.1%)	839 (96.0%)	634 (95.1%)	144 (85.2%)	525 (93.8%)
**Blood culture (positive)**	197 (12.5%)	0 (0%)	197 (22.5%)	155 (23.2%)	77 (45.6%)	154 (27.5%)

Data presented as median (interquartile range) or number (percentage) of episodes. CRP, C-reactive protein; ICU, intensive care unit; LPK, leukocyte particle concentration; NLCR, neutrophil-lymphocyte count ratio; PCT, procalcitonin.

^a^ Including episodes having possible but non-verified bacterial infections, viral infections, and non-infectious diseases.

^b^ Including all episodes fulfilling the Sepsis-2 criteria for bacterial sepsis irrespective severity (i.e., sepsis, severe sepsis, and septic shock).

^c^ Including all episodes fulfilling the Sepsis-3 criteria for bacterial sepsis irrespective severity (i.e., sepsis and septic shock).

### Diagnostic performance of the biomarkers

**Fig 4:**
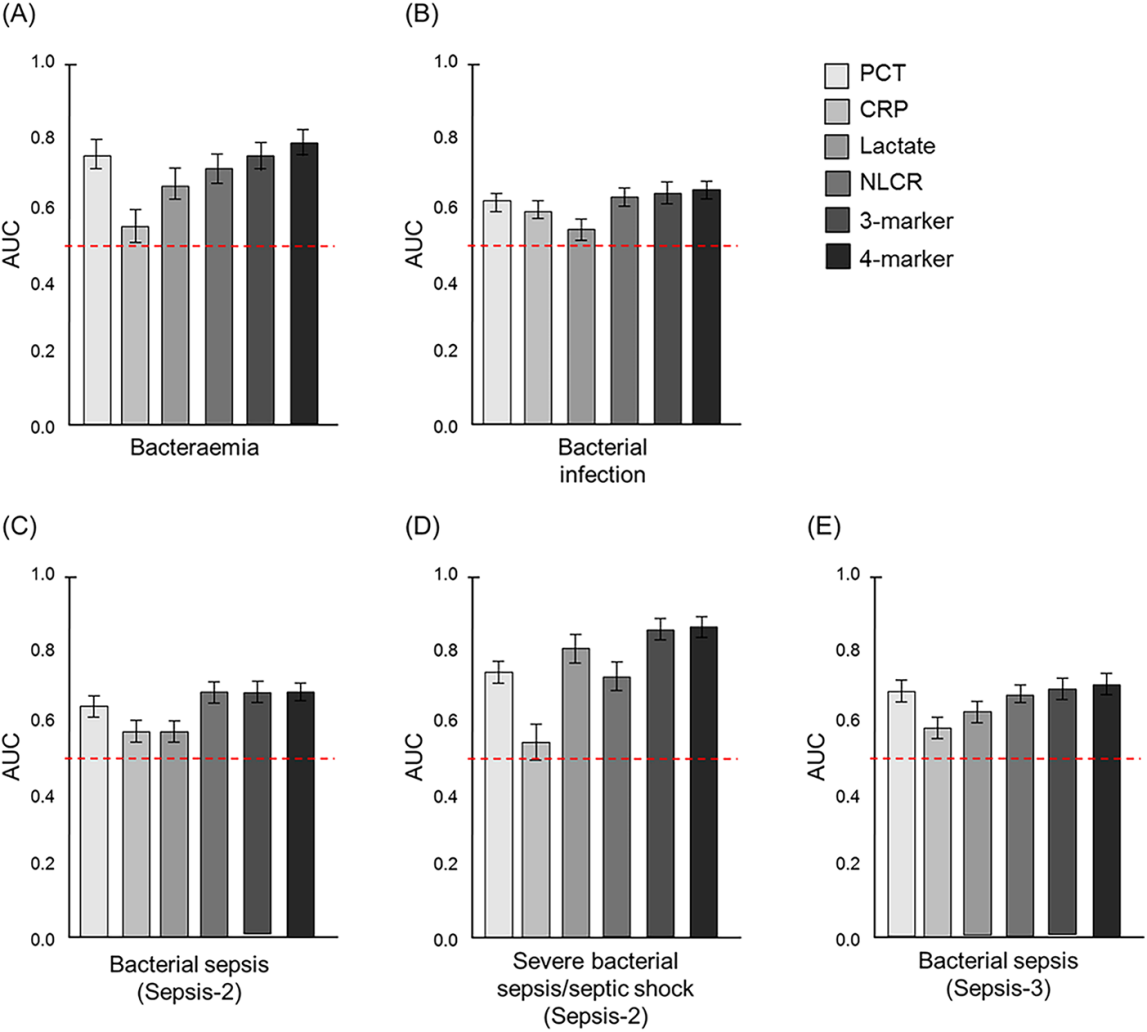
AUC for the biomarkers evaluated in the present study. Error bars represent 95% CI. The red dotted lines represent a reference line corresponding to AUC = 0.5. The three-biomarker consists of a combination of CRP, lactate, and NLCR, whereas the four-biomarker consists of a combination of PCT, CRP, lactate, and NLCR. (A) AUC for diagnosis of bacteraemia. (B) AUC for diagnosis of verified bacterial infection. (C) AUC for diagnosis of verified bacterial sepsis using Sepsis-2 criteria irrespective severity (i.e., sepsis, severe sepsis, and septic shock). (D) AUC for diagnosis of verified severe bacterial sepsis/septic shock using Sepsis-2 criteria. (E) AUC for diagnosis of verified bacterial sepsis using Sepsis-3 criteria irrespective severity (i.e., sepsis and septic shock). AUC, area under receiver operating caracteristic curve; CI, confidence interval; CRP, C reactive protein; NLCR, neutrophil-lymphocyte ratio; PCT, procalcitonin.

Levels of the various biomarkers between different patient groups were compared using Mann-Whitney U test. The biomarker levels in the 197 episodes with bacteraemia were compared with the 1,375 episodes without bacteraemia. All four biomarkers were significantly higher (all *p* < 0.01) in episodes with bacteraemia. The respective median levels were as follows: 1.70 and 0.17 ng/mL for PCT, 128.0 and 100.0 mg/L for CRP, 2.10 and 1.66 mmol/L for lactate, 16.6 and 8.8 for the NLCR (Fig 3). In the ROC curve analysis, PCT (AUC 0.74; 95% CI 0.70–0.78) showed the highest ability to diagnose bacteraemia among the single biomarkers (Fig 4A) and was significantly better than both CRP (*p* < 0.001; AUC 0.56; 95% CI 0.51–0.60) and lactate (*p* = 0.001; 0.66; 95% CI 0.61–0.70), but not the NLCR (*p* = 0.17; AUC 0.71; 95% CI 0.67–0.75). However, the composite four-biomarker had the highest AUC (0.78; 95% CI 0.74–0.81) and was significantly higher (all *p* < 0.001) than the composite three-biomarker (AUC 0.75; 95% CI 0.71–0.79) and all single biomarkers except PCT (*p* = 0.06).

The biomarker levels in the 874 episodes having a verified bacterial infection were compared with the other 698 episodes, which did either have a non-verified bacterial infection or a viral infection or a non-infectious disease. All biomarker levels were significantly higher (all *p* < 0.01) in episodes having a bacterial infection (Table 1 and Fig 3). In the ROC curve analysis (Fig 4B), the NLCR showed the highest ability among the single biomarkers to discriminate between verified bacterial infections and others (AUC 0.63; 95% CI 0.61–0.66), but was not significantly better (*p* = 0.52) than PCT (AUC 0.62; 95% CI 0.60–0.65). The AUCs for both composite biomarkers were comparable to the NLCR and PCT (all *p* > 0.05); the three-biomarker had an AUC of 0.64 (95% CI 0.62–0.67) and the four-biomarker had an AUC of 0.64 (95% CI 0.61–0.67).

The biomarker levels in the 667 episodes fulfilling the Sepsis-2 criteria for verified bacterial sepsis, including episodes ranging from sepsis to septic shock, were compared with the remaining 870 episodes excluding 35 episodes unable to determine. There were significant differences between these two groups for all four biomarkers (all *p* < 0.001). The respective median levels were as follows: 0.37 and 0.13 ng/mL for PCT, 114.0 and 91.0 mg/L for CRP, 1.81 and 1.60 mmol/L for lactate, 13.1 and 7.7 for the NLCR (Fig 3). For diagnosing bacterial sepsis, the NLCR showed a significantly higher AUC (0.68; 95% CI 0.65–0.71) than PCT (*p* = 0.019; AUC 0.64; 95% CI 0.61–0.67), CRP (*p* < 0.001; AUC 0.57; 95% CI 0.54–0.60), and lactate (*p* < 0.001; AUC 0.57; 95% CI 0.54–0.60; Fig 4C). Both composite biomarkers showed an ability equal to that of the NLCR in diagnosis of bacterial sepsis (all *p* > 0.05).

Significant differences between the 169 episodes diagnosed as either severe bacterial sepsis or septic shock based on Sepsis-2 criteria and the 1,403 episodes that did not fulfil these criteria were found for all biomarkers (*p* < 0.001), except for CRP (*p* = 0.136). The respective median levels were as follows: 1.17 and 0.17 ng/mL for PCT, 109.0 and 101.0 mg/L for CRP, 3.30 and 1.61 mmol/L for lactate, 18.4 and 8.9 for the NLCR (Fig 3). When comparing the AUC in severe bacterial sepsis/septic shock for the biomarkers (Fig 4D), lactate had the highest AUC (0.81; 95% CI 0.77–0.85) among the single biomarkers and was significantly better than PCT (*p* = 0.01), the NLCR (*p* < 0.01) and CRP (*p* < 0.001). The composite three-biomarker (AUC 0.86; 95% CI 0.83–0.89) as well as the four-biomarker (0.85; 95% CI 0.82–0.88) were both significantly better than all single biomarkers (all *p* <0.05).

The 560 episodes fulfilling the Sepsis-3 criteria for verified bacterial sepsis were compared to the 1,012 episodes not fulfilling these criteria, and significant differences were found for all biomarkers (all *p* < 0.01). The respective median levels were as follows: 0.51 and 0.13 ng/mL for PCT, 126.0 and 91.0 mg/L for CRP, 1.90 and 1.60 mmol/L for lactate, 13.0 and 8.0 for the NLCR. In the ROC curve analysis (Fig 4E), PCT showed the highest ability among the single biomarkers to discriminate between episodes with verified bacterial sepsis and episodes not having a verified bacterial sepsis (AUC 0.68; 95% CI 0.65–0.71), but was not significantly better (*p* = 0.35) than the NLCR (AUC 0.67; 95% CI 0.64–0.69). The AUCs for both composite biomarkers were comparable to PCT (both *p* > 0.05); the three-biomarker had an AUC of 0.69 (95% CI 0.66–0.72) and the four-biomarker had an AUC of 0.70 (95% CI 0.67–0.73).

The computed specificities, sensitivities, accuracy, diagnostic odds ratios, as well as positive and negative predictive values of the single biomarkers at selected cut-off values for diagnosis of verified bacterial sepsis using Sepsis-2 criteria are shown in Table 2; for diagnosis of verified severe bacterial sepsis/septic shock using Sepsis-2 criteria are shown in Table 3; for diagnosis of verified bacterial sepsis using Sepsis-3 criteria are shown in Table 4. Complete performance characteristics of the single biomarkers at additional cut-off values are presented in Supporting Information (S1–S3 Tables).

**Table 2:** Performance characteristics of single biomarkers for diagnosing verified bacterial sepsis using Sepsis-2 criteria^a^.

Biomarker (cut-off)	Sensitivity (95% CI)	Specificity (95% CI)	Accuracy (95% CI)	DOR (95% CI)	PPV (95% CI)	NPV (95% CI)
**PCT (2.0 ng/mL)**	26.4% (23.0–29.7)	88.6% (86.5–90.7)	61.6% (59.2–64.0)	2.79 (2.13–3.66)	64.0% (58.3–70.0)	61.0% (58.4–63.8)
**PCT (10.0 ng/mL)**	11.1% (8.7–13.5)	96.4% (95.2–97.7)	59.4 (57.0–61.9)	3.38 (2.19–5.20)	70.5% (61.8–79.2)	58.6% (56.0–61.1)
**CRP (20 mg/L)**	88.1% (85.7–90.6)	14.5% (12.1–16.8)	46.4% (43.9–49.0)	1.26 (0.93–1.70)	44.1% (41.4–46.8)	61.4% (54.7–68.1)
**CRP (100 mg/L)**	57.1% (53.3–60.9)	52.3% (49.9–56.6)	54.9% (52.4–57.4)	1.51 (1.23–1.86)	48.3% (44.8–51.8)	61.8% (58.3–65.3)
**Lactate (2.5 mmol/L)**	24.9% (21.5–28.2)	82.7% (80.1–85.2)	57.4% (54.9–59.9)	1.58 (1.22–2.03)	52.6% (47.0–58.2)	58.7% (55.9–61.5)
**Lactate (3.5 mmol/L)**	11.9% (9.4–14.4)	94.4% (92.8–95.9)	58.4% (55.9–60.9)	4.84 (2.69–8.71)	62.1% (53.6–70.6)	58.0% (55.4–60.6)
**NLCR (3.0)**	95.9% (94.4–97.4)	13.2% (10.9–15.5)	49.0% (46.5–51.6)	3.54 (2.29–5.45)	45.8% (43.2–48.4)	80.7% (74.2–87.3)
**NLCR (10.0)**	64.3% (60.6–67.9)	64.0% (60.8–67.2)	64.1% (61.7–66.6)	3.20 (2.59–3.96)	57.8% (54.2–61.3)	70.1% (66.9–73.3)

CRP, C-reactive protein; DOR, diagnostic odds ratio; NLCR, neutrophil-lymphocyte count ratio; NPV, negative predictive value; PCT, procalcitonin; PPV, predictive positive value.

^a^Including all episodes fulfilling the Sepsis-2 criteria for bacterial sepsis irrespective severity (i.e., sepsis, severe sepsis, and septic shock).

**Table 3:** Performance characteristics of single biomarkers for diagnosing verified severe bacterial sepsis/bacterial septic shock using Sepsis-2 criteria.

Biomarker (cut-off)	Sensitivity (95% CI)	Specificity (95% CI)	Accuracy (95% CI)	DOR (95% CI)	PPV (95% CI)	NPV (95% CI)
**PCT (2.0 ng/mL)**	46.8% (39.2–54.3)	85.5% (83.7–87.4)	81.3% (79.4–83.3)	5.19 (3.70–7.27)	28.1% (22.9–33.4)	92.3% (91.6–94.4)
**PCT (10.0 ng/mL)**	26.6% (20.0–33.3)	95.5% (94.4–96.6)	88.1% (86.4–89.7)	7.68 (5.02–1.74)	41.7% (32.4–51.0)	91.5% (90.1–92.9)
**CRP (20 mg/L)**	87.3% (82.2–92.4)	13.5% (11.7–15.3)	21.4% (19.4–23.5)	1.07 (0.66–1.74)	10.8% (9.1–12.5)	89.9% (85.7–94.0)
**CRP (100 mg/L)**	56.4% (48.8–63.9)	49.4% (46.7–52.0)	50.1% (47.6–52.6)	1.26 (0.91–1.74)	11.8% (9.5–14.0)	90.4% (88.3–92.5)
**Lactate (2.5 mmol/L)**	66.5% (59.2–73.7)	85.1% (83.2–87.0)	83.1% (81.2–85.0)	11.36 (7.94–16.23)	35.3% (30.0–40.6)	95.4% (94.2–96.6)
**Lactate (3.5 mmol/L)**	67.1% (61.4–72.7)	96.5% (95.5–97.5)	91.7% (90.3–93.0)	56.23 (38.15–82.89)	79.0% (73.7–84.4)	93.7% (92.5–95.0)
**NLCR (3.0)**	96.4% (93.6–99.2)	10.0% (8.4–11.6)	19.4% (17.4–21.3)	2.98 (1.29–6.95)	11.5% (9.9–13.2)	95.8% (92.5–99.1)
**NLCR (10.0)**	79.0% (72.9–85.2)	55.6% (53.0–58.2)	58.2% (55.7–60.6)	4.73 (3.21–6.96)	17.8% (15.1–20.6)	95.6% (94.2–97.0)

CRP, C-reactive protein; DOR, diagnostic odds ratio; NLCR, neutrophil-lymphocyte count ratio; NPV, negative predictive value; PCT, procalcitonin; PPV, predictive positive value.

**Table 4:** Performance characteristics of single biomarkers for diagnosing verified bacterial sepsis using Sepsis-3 criteria^a^.

Biomarker (cut-off)	Sensitivity (95% CI)	Specificity (95% CI)	Accuracy (95% CI)	DOR (95% CI)	PPV (95% CI)	NPV (95% CI)
**PCT (2.0 ng/mL)**	32.1% (28.3–36.0)	89.5% (87.6–91.4)	69.1% (66.8–71.4)	4.05 (3.10–5.29)	62.9% (57.3–68.5)	70.5% (68.0–72.9)
**PCT (10.0 ng/mL)**	15.4% (12.4–18.3)	97.4% (96.5–98.4)	68.2% (65.9–70.5)	6.88 (4.38–10.81)	76.8% (69.0–84.6)	67.5% (65.1–69.9)
**CRP (20 mg/L)**	88.4% (85.8–91.1)	14.4% (12.2–16.5)	40.8% (38.4–43.3)	1.28 (0.93–1.75)	36.4% (33.9–39.0)	69.0% (62.8–75.4)
**CRP (100 mg/L)**	59.7% (55.6–63.8)	53.3% (50.2–56.4)	55.6% (53.1–58.1)	1.69 (1.37–2.09)	41.5% (38.1–44.9)	70.4% (67.2–73.7)
**Lactate (2.5 mmol/L)**	29.5% (25.7–33.4)	84.2% (81.9–86.5)	64.6% (62.2–67.1)	2.23 (1.73–2.87)	51.0% (45.4–56.5)	68.2% (65.6–70.9)
**Lactate (3.5 mmol/L)**	14.9% (11.9–18.0)	95.3% (94.0–96.6)	66.6% (64.2–68.9)	3.54 (2.43–5.18)	63.8% (55.4–72.1)	66.8% (64.3–69.3)
**NLCR (3.0)**	95.1% (93.3–96.9)	11.7% (9.7–13.7)	41.5% (39.1–44.0)	2.58 (1.67–3.97)	37.5% (35.0–40.0)	81.1% (74.7–87.5)
**NLCR (10.0)**	64.7% (60.8–68.7)	60.8% (57.9–63.9)	62.2% (59.8–64.6)	2.85 (2.30–3.54)	47.9% (44.3–51.5)	75.6% (72.6–78.6)

CRP, C-reactive protein; DOR, diagnostic odds ratio; NLCR, neutrophil-lymphocyte count ratio; NPV, negative predictive value; PCT, procalcitonin; PPV, predictive positive value.

^a^Including all episodes fulfilling the Sepsis-3 criteria for bacterial sepsis irrespective severity (i.e., sepsis and septic shock).

## Discussion

Over the years, numerous studies have been performed investigating the clinical usefulness of biomarkers in diagnosis, prognosis, staging, and monitoring of sepsis [34, 56, 57]. Many studies have thus focused on the use of single biomarkers although the interest in multimarker approaches in sepsis diagnostics has increased, especially in the search for novel sepsis biomarkers using high-throughput methods for screening of patient samples [58–61]. A weakness in many previous biomarkers studies is the use of small sample size of patients which may lead to inconsistent results [56]. In the present study, we have investigated the diagnostic value of known biomarkers, i.e., PCT, the NLCR, CRP and lactate, alone as well as in combination using a large sample consisting of 1,572 episodes of adult patients suspected with sepsis.

Recently, the criteria for sepsis were updated [1] and the definition of sepsis was changed from a systemic inflammatory response syndrome caused by an infection (Sepsis-2) to a life-threatening organ dysfunction due to a dysregulated host response to infection (Sepsis-3). Changing the criteria may ultimately also affect the performance of diagnostic biomarkers. In the present study, we found that CRP at a cut-off of 20 mg/mL had the highest sensitivity (88%) for bacterial sepsis as defined by Sepsis-3 criteria, whereas both PCT at a cut-off 10.0 ng/mL and lactate at a cut-off of 4.0 mmol/L showed high specificity (97%). Considering the AUCs, all single biomarkers except for the NLCR performed slightly better in diagnosing bacterial sepsis based on Sepsis-3 criteria than Sepsis-2 criteria (Fig 4). PCT showed a moderate AUC (0.68), but was nonetheless the single biomarker with the highest AUC and comparable to the AUCs for the composite variables (Fig 4E). However, more studies based on the updated Sepsis-3 criteria are needed to further assess the diagnostic performance of these biomarkers.

The NLCR has been reported to correlate with the severity of disease [41–43] and has gained interest as a predictor of survival in various clinical circumstances ranging from oncological patients to patients with cardiovascular diseases [62–67]. In the context of sepsis, the NLCR has previously been described as a predictor of bacteraemia [44–48]. In this study, the AUC for predicting positive blood culture results based on the NLCR was moderate (0.71) and in line with previous studies reporting AUCs of 0.73 [44] and 0.77 [48]. In the ROC curve analysis for detection of bacterial sepsis based on Sepsis-2 criteria, the NLCR showed a moderate AUC (0.68), nonetheless significantly higher than all the other single biomarkers (Fig 4C). The diagnostic discriminatory power is usually improved when combining information from several biomarkers [68, 69], but here it can be observed that the NLCR had an ability equal to both composite biomarkers for diagnosing bacterial sepsis. The NLCR also demonstrated a high sensitivity (96%, Tables 2 and 3) at a cut-off of 3.0 for diagnosis of bacterial sepsis as well as severe bacterial sepsis or septic shock using Sepsis-2 criteria. This suggests that low NLCR values (<3.0) can be used to exclude bacteria as the etiological cause in critically ill patients.

PCT has been investigated in many studies previously and the outcomes have been contradicting [20–38]. One reason could be that many studies have had relatively small sample sizes [70–72] which may undermine the reliability of the results [73]. In addition, only a minority of the previous studies have formally excluded patients treated with antibiotics before inclusion. This may result in false-negative results due to possible antibiotic treatment before inclusion, leading to underestimation of the effect. Besides small sample sizes and antibiotic treatment before inclusion, other factors such as differences in target population, type of clinical setting, cut-off value, disease prevalence, assay type, and disease severity could also have contributed to the conflicting results in previous studies. In the present study, PCT showed a high specificity (96%, Tables 2 and 3) at a cut-off of 10.0 ng/mL for diagnosis of bacterial sepsis and severe bacterial sepsis or septic shock using Sepsis-2 criteria. This means that patients with a PCT concentration of 10 ng/mL or higher, are likely to have bacterial sepsis. The numerous factors that may affect the diagnostic performance of a biomarker and thereby contributing to contradicting results also make it difficult to directly compare outcomes between studies. However, the sensitivities for PCT in sepsis diagnostics described in the present study (Table 2) are generally lower compared with previous findings [35, 37]. This is consistent with the reflection made in a review by Kibe *et al*., who noted that larger studies tend to find lower estimates of PCT sensitivity than smaller studies [73]. For instance, using a cut-off of 0.5 ng/mL for PCT generated a sensitivity of 44% (S1 Table) whereas the sensitivities reported by others usually are in the range 60–90% [74–78]. Also the sensitivity of 26% at a cut-off of 2.0 ng/mL (S1 Table) is considerably lower than described in other studies where the sensitivity typically ranges between 65% and 97% [71, 79, 80].

The results from the present study suggest lactate as the single best-performing biomarker in diagnosing severe bacterial sepsis and septic shock based on Sepsis-2 criteria (Table 3). Using a cut-off of 3.5 mmol/L for lactate results in a specificity of 97%, an accuracy of 92% and a DOR of 56.23. This result is not surprising as lactate >3.5 mmol/L is included in the criteria for severe sepsis (S1 Text) and might therefore be due to incorporation bias. Although lactate is widely used for early detection of sepsis, elevated levels of lactate are not considered specific for diagnosis of sepsis [40]. Lactate has proven more valuable as prognostic sepsis biomarker as elevated lactate levels have been associated with high mortality in several studies [81–83]. Both composite biomarkers had significantly higher AUCs than all the single biomarkers including lactate in diagnosing severe bacterial sepsis and septic shock (Fig 4D) which further suggest the need of a joint interpretation of several biomarkers in sepsis diagnostics.

The present study demonstrate that combinations of biomarkers appears to be a useful approach to improve the diagnostic accuracy for bacterial sepsis, irrespectively of using Sepsis-2 or Sepsis-3 criteria (Fig 4). For all diagnostic categories investigated, except for bacterial sepsis defined according Sepsis-2 criteria, the four-biomarker had a higher AUC than all single biomarkers. A major challenge in using multimarker approaches is the translation of several measurements into one composite variable. In this study, we employed different methods (e.g., linear discriminant analysis, logistic regression, and additive composite variable based on normalized values) to combine several biomarkers into one variable. As the diagnostic ability were found to be equivalent for all three types of composite variables, we chose to only report the results of those composite biomarkers developed using linear discriminant analysis as a linear combination of variable can easily be adopted for clinical use. Other drawbacks of multimarker approaches may include high cost and less availability in comparison with single biomarker as the NLCR for example, and thus limiting their utility, especially in resource-limited settings. In the future we foresee that much more complex multimarker combinations will be developed for diagnostic purposes and thus will probably require digital support for the interpretation.

Several limitations of this study merit consideration. First, there is no gold standard test by which to diagnose bacterial sepsis. We therefore used a strict definition of bacterial sepsis and only included episodes with verified bacterial infection, i.e., either clinical infection with a positive culture or typical clinical symptoms such as erysipelas. However, many patients do have bacterial infections without being microbiologically proven. So most probably a part of the episodes categorised as ‘others’, i.e., non-verified bacterial infections, viral infections and no infections, had a bacterial infection and this may have negatively affected the diagnostic performance. Secondly, this study was performed prior the introduction of the new sepsis definition, Sepsis-3 [1]. We assessed the diagnostic accuracy of the biomarkers using this new definition as well, but were only able to assess it for the diagnosis of bacterial sepsis, not bacterial septic shock, based on available data. Finally, it is probably an oversimplification to use linear models for combining biomarkers. The diagnostic accuracy might be further improved by using non-linear approaches.

## Supporting information

S1 Text Swedish criteria (2011) for hypotension, hypoperfusion and organ dysfunction in severe sepsis and septic shock in adults. (PDF)

S2 Text Linear discriminant analysis. (PDF)

S1 Table Performance characteristics of single biomarkers at different cut-offs for diagnosing verified bacterial sepsis using Sepsis-2 criteria. (PDF)

S2 Table Performance characteristics of single biomarkers at different cut-offs for diagnosing verified severe bacterial sepsis/bacterial septic shock using Sepsis-2 criteria. (PDF)

S3 Table Performance characteristics of single biomarkers at different cut-offs for diagnosing verified bacterial sepsis using Sepsis-3 criteria. (PDF)
