# Supplementary material for: Diagnostic accuracy of procalcitonin, neutrophil-lymphocyte count ratio, C-reactive protein, and lactate in patients with suspected bacterial sepsis
Source: PLoS One. 2017 Jul 20;12(7):e0181704. doi: 10.1371/journal.pone.0181704 (PMC5519182; doi:10.1371/journal.pone.0181704)
Supplement: S1 Text — (PDF) [file pone.0181704.s001.pdf]

S1 Text. Swedish criteria (2011) for hypotension, hypoperfusion and organ dysfunction in severe sepsis and septic shock in adults

Hypotension

Systolic blood pressure (SBP) <90 mmHg

Hypoperfusion

P-lactate >3.5mmol/L

Organ dysfunction

At least one of these:

- Renal dysfunction: Oliguria <0.5 ml/kg per hour for two hours despite fluid resuscitation
- Hepatic dysfunction: P-bilirubin >70µmol/L
- Coagulopathy: Platelet count <100 x10^9/L or international normalized ratio (INR) >1.5 or activated partial thromboplastin time (aPTT) >60s
- Neurologic dysfunction: Altered mental status
- Respiratory dysfunction: PaO_2/FiO_2 <250 or oxygen saturation <87%; if the lung is the source of infection, PaO_2/FiO_2 <200 or oxygen saturation <79%
