# Supplementary material for: Diagnostic accuracy of procalcitonin, neutrophil-lymphocyte count ratio, C-reactive protein, and lactate in patients with suspected bacterial sepsis
Source: PLoS One. 2017 Jul 20;12(7):e0181704. doi: 10.1371/journal.pone.0181704 (PMC5519182; doi:10.1371/journal.pone.0181704)
Supplement: S2 Text — (PDF) [file pone.0181704.s002.pdf]

S2 Text. Linear discriminant analysis

Linear discriminant analysis (LDA) is a commonly used multivariate classification method. The objective of LDA is to develop discriminant functions that are linear combinations of independent variables that will maximize the discrimination between the categories of the dependent variable. It is simple, mathematically robust and often produce models whose accuracy is as good as more complex methods. We constructed two discriminant functions, one based on three independent variables (i.e., the neutrophil-lymphocyte count ratio, C-reactive protein, and lactate) and one based on four independent variables (i.e., procalcitonin, the neutrophil-lymphocyte count ratio, C-reactive protein, and lactate), using IBM SPSS Statistics version 24.0 (IBM Corp., United States) for each of the dependent variable (i.e., bacteraemia vs. non-bacteraemia, verified bacterial infection vs. others, verified bacterial sepsis (Sepsis-2) vs. no bacterial sepsis (Sepsis-2), verified severe bacterial sepsis/septic shock (Sepsis-2) vs. no severe bacterial sepsis/septic shock (Sepsis-2), verified bacterial sepsis (Sepsis-3) vs. no bacterial sepsis (Sepsis-3)). Each constructed discriminant function consisted of a linear equation of the original independent variables:

$$Z = \beta_1 X_1 + \beta_2 X_2 + \ \dots + \beta_n X_n$$

where Z is the discriminant score, the β's are the discriminant coefficients, and X's are the independent variables. The discriminant score Z was computed for each episode and used to predict category of the dependent variable as well to compute the area under the receiver operating characteristic curve (AUC).
