# Supplementary material for: Diagnostic accuracy of procalcitonin, neutrophil-lymphocyte count ratio, C-reactive protein, and lactate in patients with suspected bacterial sepsis
Source: PLoS One. 2017 Jul 20;12(7):e0181704. doi: 10.1371/journal.pone.0181704 (PMC5519182; doi:10.1371/journal.pone.0181704)
Supplement: S1 Table — (PDF) [file pone.0181704.s003.pdf]

S1 Table. Performance characteristics of single biomarkers at different cut-offs for diagnosing verified bacterial sepsis using Sepsis-2 criteria.[a]

Biomarker (cut-off)	Sensitivity (95% CI)	Specificity (95% CI)	Accuracy (95% CI)	DOR (95% CI)	PPV (95% CI)	NPV (95% CI)
**PCT (0.1 ng/ml)**	76.0% (72.7-79.3)	42.0% (38.7-45.2)	56.7% (54.3-59.2)	2.29 (1.83-2.86)	50.1% (47.0-53.2)	69.5% (65.6-73.5)
**PCT (0.5 ng/mL)**	44.4% (40.6-48.2)	73.7 % (70.8-76.6)	61.0% (58.5-63.4)	2.23 (1.80-2.77)	56.4% (52.1-60.6)	63.3% (60.4-66.3)
**PCT (2.0 ng/mL)**	26.4% (23.0-29.7)	88.6% (86.5-90.7)	61.6% (59.2-64.0)	2.79 (2.13-3.66)	64.0% (58.3-70.0)	61.0% (58.4-63.8)
**PCT (10.0 ng/mL)**	11.1% (8.7-13.5)	96.4% (95.2-97.7)	59.4 (57.0-61.9)	3.38 (2.19-5.20)	70.5% (61.8-79.2)	58.6% (56.0-61.1)
**CRP (20 mg/L)**	88.1% (85.7-90.6)	14.5% (12.1-16.8)	46.4% (43.9-49.0)	1.26 (0.93-1.70)	44.1% (41.4-46.8)	61.4% (54.7-68.1)
**CRP (50 mg/L)**	75.7% (72.4-78.9)	32.0% (28.9-35.1)	50.9% (48.4-53.4)	1.46 (1.16-1.84)	46.0% (43.1-49.0)	63.1% (58.6-67.7)
**CRP (100 mg/L)**	57.1% (53.3-60.9)	52.3% (49.9-56.6)	54.9% (52.4-57.4)	1.51 (1.23-1.86)	48.3% (44.8-51.8)	61.8% (58.3-65.3)
**Lactate (2.0 mmol/L**	42.1% (38.3-45.9)	68.8% (65.6-71.9)	57.1% (54.6-60.0)	1.60 (1.30-1.99)	51.1% (46.9-55.4)	60.5% (57.4-63.6)
**Lactate (2.5 mmol/L)**	24.9% (21.5-28.2)	82.7% (80.1-85.2)	57.4% (54.9-59.9)	1.58 (1.22-2.03)	52.6% (47.0-58.2)	58.7% (55.9-61.5)
**Lactate (3.5 mmol/L)**	11.9% (9.4-14.4)	94.4% (92.8-95.9)	58.4% (55.9-60.9)	4.84 (2.69-8.71)	62.1% (53.6-70.6)	58.0% (55.4-60.6)
**Lactate (4.0 mmol/L**	7.4% (5.4-9.4)	96.2% (94.9-97.5)	57.4% (54.9-59.9)	2.01 (1.27-3.18)	60.0% (49.3-70.7)	57.3% (54.7-59.9)
**NLCR (3.0)**	95.9% (94.4-97.4)	13.2% (10.9-15.5)	49.0% (46.5-51.6)	3.54 (2.29-5.45)	45.8% (43.2-48.4)	80.7% (74.2-87.3)
**NLCR (10.0)**	64.3% (60.6-67.9)	64.0% (60.8-67.2)	64.1% (61.7-66.6)	3.20 (2.59-3.96)	57.8% (54.2-61.3)	70.1% (66.9-73.3)

**NLCR (12.0)**	54.7% (50.8-58.5)	71.7% (68.7-74.8)	64.3% (61.9-66.7)	3.06 (2.47-3.79)	59.7% (55.7-63.6)	67.4% (64.4-70.4)
**NLCR (15.0)**	44.1% (40.3-47.9)	79.0% (76.4-81.8)	63.9% (61.5-66.4)	2.99 (2.38-3.74)	61.8% (57.4-66.2)	64.9% (62.0-67.8)
**NLCR (20.0)**	29.0% (25.5-32.5)	88.1% (85.9-90.3)	62.5% (60.0-64.9)	3.02 (2.31-3.94)	65.1% (59.6-70.5)	61.9% (59.1-64.6)

CRP, C-reactive protein; DOR, diagnostic odds ratio; NLCR, neutrophil-lymphocyte count ratio; NPV, negative predictive value; PCT, procalcitonin; PPV, predictive positive value.

[a]Including all episodes fulfilling the Sepsis-2 criteria for bacterial sepsis irrespective severity (i.e., sepsis, severe sepsis, and septic shock).
