# Supplementary material for: Diagnostic accuracy of procalcitonin, neutrophil-lymphocyte count ratio, C-reactive protein, and lactate in patients with suspected bacterial sepsis
Source: PLoS One. 2017 Jul 20;12(7):e0181704. doi: 10.1371/journal.pone.0181704 (PMC5519182; doi:10.1371/journal.pone.0181704)
Supplement: S2 Table — (PDF) [file pone.0181704.s004.pdf]

S2 Table. Performance characteristics of single biomarkers at different cut-offs for diagnosing verified severe bacterial sepsis/bacterial septic shock using Sepsis-2 criteria.

Biomarker (cut-off)	Sensitivity (95% CI)	Specificity (95% CI)	Accuracy (95% CI)	DOR (95% CI)	PPV (95% CI)	NPV (95% CI)
**PCT (0.1 ng/mL)**	87.6% (82.6-92.6)	37.0% (34.4-39.5)	42.4% (40.0-44.9)	4.13 (2.58-6.61)	14.4% (12.3-16.5)	96.1% (94.5-97.7)
**PCT (0.5 ng/mL)**	61.5% (54.2-68.9)	69.1% (66.6-71.5)	68.2% (65.9-70.6)	3.57 (2.57-4.97)	19.4% (16.1-22.8)	93.7% (92.2-95.2)
**PCT (2.0 ng/mL)**	46.8% (39.2-54.3)	85.5% (83.7-87.4)	81.3% (79.4-83.3)	5.19 (3.70-7.27)	28.1% (22.9-33.4)	92.3% (91.6-94.4)
**PCT (10.0 ng/mL)**	26.6% (20.0-33.3)	95.5% (94.4-96.6)	88.1% (86.4-89.7)	7.68 (5.02-1.74)	41.7% (32.4-51.0)	91.5% (90.1-92.9)
**CRP (20 mg/L)**	87.3% (82.2-92.4)	13.5% (11.7-15.3)	21.4% (19.4-23.5)	1.07 (0.66-1.74)	10.8% (9.1-12.5)	89.9% (85.7-94.0)
**CRP (50 mg/L)**	72.1% (65.3-79.0)	28.8% (26.4-31.2)	33.4% (31.0-35.8)	1.04 (0.73-1.50)	10.8% (9.0-12.7)	89.6% (86.8-92.4)
**CRP (100 mg/L)**	56.4% (48.8-63.9)	49.4% (46.7-52.0)	50.1% (47.6-52.6)	1.26 (0.91-1.74)	11.8% (9.5-14.0)	90.4% (88.3-92.5)
**Lactate (2.0 mmol/L**	76.8% (70.4-83.3)	69.3% (66.9-71.8)	70.1% (67.8-72.4)	7.49 (5.12-10.96)	23.4% (19.8-27.0)	96.1% (94.9-97.3)
**Lactate (2.5 mmol/L)**	66.5% (59.2-73.7)	85.1% (83.2-87.0)	83.1% (81.2-85.0)	11.36 (7.94-16.23)	35.3% (30.0-40.6)	95.4% (94.2-96.6)
**Lactate (3.5 mmol/L)**	67.1% (61.4-72.7)	96.5% (95.5-97.5)	91.7% (90.3-93.0)	56.23 (38.15-82.89)	79.0% (73.7-84.4)	93.7% (92.5-95.0)
**Lactate (4.0 mmol/L**	29.3% (22.3-36.2)	97.6% (96.8-98.4)	90.2% (88.7-91.7)	16.99 (10.45-27.62)	60.0% (49.3-70.7)	91.9% (90.5-93.3)
**NLCR (3.0)**	96.4% (93.6-99.2)	10.0% (8.4-11.6)	19.4% (17.4-21.3)	2.98 (1.29-6.95)	11.5% (9.9-13.2)	95.8% (92.5-99.1)
**NLCR (10.0)**	79.0% (72.9-85.2)	55.6% (53.0-58.2)	58.2% (55.7-60.6)	4.73 (3.21-6.96)	17.8% (15.1-20.6)	95.6% (94.2-97.0)
**NLCR (12.0)**	71.9% (65.0-78.7)	64.4% (61.8-66.9)	65.2% (62.8-67.6)	4.61 (3.23-6.57)	19.7% (16.6-22.9)	95.0% (93.5-96.4)
**NLCR**	62.3%	73.0%	71.9%	4.47 (3.20-	21.9%	94.1%

**(15.0)**	(54.9-69.6)	(70.7-75.4)	(69.6-74.1)	6.25)	(18.2-25.7)	(92.7-95.5)
**NLCR (20.0)**	43.7% (36.2-51.2)	83.8% (81.8-85.7)	79.4% (77.4-81.4)	4.00 (2.85-5.61)	24.7% (19.8-29.6)	92.4% (91.0-93.9)

CRP, C-reactive protein; DOR, diagnostic odds ratio; NLCR, neutrophil-lymphocyte count ratio; NPV, negative predictive value; PCT, procalcitonin; PPV, predictive positive value.
