# Supplementary material for: Diagnostic accuracy of procalcitonin, neutrophil-lymphocyte count ratio, C-reactive protein, and lactate in patients with suspected bacterial sepsis
Source: PLoS One. 2017 Jul 20;12(7):e0181704. doi: 10.1371/journal.pone.0181704 (PMC5519182; doi:10.1371/journal.pone.0181704)
Supplement: S3 Table — (PDF) [file pone.0181704.s005.pdf]

S3 Table. Performance characteristics of single biomarkers at different cut-offs for diagnosing verified bacterial sepsis using Sepsis-3 criteria.[a]

Biomarker (cut-off)	Sensitivity (95% CI)	Specificity (95% CI)	Accuracy (95% CI)	DOR (95% CI)	PPV (95% CI)	NPV (95% CI)
**PCT (0.1 ng/mL)**	79.8% (76.5-83.2)	41.9% (38.9-44.9)	55.4% (53.0-57.9)	2.85 (2.24-3.63)	43.2% (40.2-46.2)	79.0% (75.5-82.4)
**PCT (0.5 ng/mL)**	50.4% (46.2-54.5)	74.2% (71.5-76.9)	65.7% (63.4-68.1)	2.92 (2.35-3.63)	51.9% (47.7-56.1)	73.0% (70.3-75.7)
**PCT (2.0 ng/mL)**	32.1% (28.3-36.0)	89.5% (87.6-91.4)	69.1% (66.8-71.4)	4.05 (3.10-5.29)	62.9% (57.3-68.5)	70.5% (68.0-72.9)
**PCT (10.0 ng/mL)**	15.4% (12.4-18.3)	97.4% (96.5-98.4)	68.2% (65.9-70.5)	6.88 (4.38-10.81)	76.8% (69.0-84.6)	67.5% (65.1-69.9)
**CRP (20 mg/L)**	88.4% (85.8-91.1)	14.4% (12.2-16.5)	40.8% (38.4-43.3)	1.28 (0.93-1.75)	36.4% (33.9-39.0)	69.0% (62.8-75.4)
**CRP (50 mg/L)**	76.3% (72.7-79.9)	31.3% (28.4-34.2)	47.4% (44.9-49.9)	1.47 (1.16-1.86)	38.2% (35.3-41.0)	70.4% (66.2-74.7)
**CRP (100 mg/L)**	59.7% (55.6-63.8)	53.3% (50.2-56.4)	55.6% (53.1-58.1)	1.69 (1.37-2.09)	41.5% (38.1-44.9)	70.4% (67.2-73.7)
**Lactate (2.0 mmol/L)**	45.6% (41.4-49.8)	69.5% (66.6-72.4)	61.0% (58.5-63.4)	1.91 (1.54-2.37)	45.4% (41.2-49.6)	69.7% (66.8-72.5)
**Lactate (2.5 mmol/L)**	29.5% (25.7-33.4)	84.2% (81.9-86.5)	64.6% (62.2-67.1)	2.23 (1.73-2.87)	51.0% (45.4-56.5)	68.2% (65.6-70.9)
**Lactate (3.5 mmol/L)**	14.9% (11.9-18.0)	95.3% (94.0-96.6)	66.6% (64.2-68.9)	3.54 (2.43-5.18)	63.8% (55.4-72.1)	66.8% (64.3-69.3)
**Lactate (4.0 mmol/L)**	9.8% (7.3-12.3)	97.0% (96.0-98.1)	65.8% (63.4-68.2)	3.53 (2.22-5.63)	64.6% (54.3-75.0)	65.9% (63.5-68.4)
**NLCR (3.0)**	95.1% (93.3-96.9)	11.7% (9.7-13.7)	41.5% (39.1-44.0)	2.58 (1.67-3.97)	37.5% (35.0-40.0)	81.1% (74.7-87.5)
**NLCR (10.0)**	64.7% (60.8-68.7)	60.8% (57.9-63.9)	62.2% (59.8-64.6)	2.85 (2.30-3.54)	47.9% (44.3-51.5)	75.6% (72.6-78.6)
**NLCR (12.0)**	55.3% (51.2-59.5)	69.1% (66.2-72.0)	64.2% (61.8-66.6)	2.77 (2.23-3.43)	49.9% (46.0-53.9)	73.5% (70.7-76.4)
**NLCR**	43.9%	76.4%	64.8%	2.54 (2.03-	50.9%	71.0%

**(15.0)**	(39.8-48.1)	(73.8-79.1)	(62.4-67.2)	3.18)	(46.4-55.4)	(68.3-73.7)
**NLCR (20.0)**	30.4% (26.6-34.2)	86.9% (84.8-89.0)	66.7% (64.3-69.0)	2.90 (2.24-3.75)	56.4% (50.8-62.0)	69.2% (66.6-71.7)

CRP, C-reactive protein; DOR, diagnostic odds ratio; NLCR, neutrophil-lymphocyte count ratio; NPV, negative predictive value; PCT, procalcitonin; PPV, predictive positive value.

[a]Including all episodes fulfilling the Sepsis-3 criteria for bacterial sepsis irrespective severity (i.e., sepsis and septic shock).
